# Normative data of corneal diameter and palpebral fissure height in a large cohort of South Indian children

**DOI:** 10.25122/jml-2023-0535

**Published:** 2024-04

**Authors:** Naveen Kumar Challa, Deepthi Jagadeeswaran, Saif Hasan Alrasheed, Abd Elaziz Mohamed Elmadina, Waleed Alghamdi

**Affiliations:** 1 Department of Optometry, College of Applied Medical Sciences, Qassim University, Buraidah, Saudi Arabia; 2 Lotus Eye Hospital and Institute, Coimbatore, India

**Keywords:** horizontal visible iris diameter, vertical visible iris diameter, palpebral fissure height, child, South India

## Abstract

The purpose of this study was to establish the normative data of horizontal visible iris diameter (HVID), vertical visible iris diameter (VVID), and palpebral fissure height (PFH) in a cohort of South Indian children. The study included 1,234 children from six schools of different regions of Tamil Nadu state, India. HVID, VVID, and PFH were measured using a simple millimeter ruler by three optometrists. Based on their age, the children were divided into three groups: preprimary school children (4–5 years), primary school children (6–10 years), and high school children (11–15 years). Mean age was 4.49 ± 0.50 years, 8.00 ± 1.41 years, and 12.87 ± 1.42 years in the three groups, respectively. Mean HVID was 10.45 mm, 10.54 mm, and 10.73 mm, respectively. Mean VVID was 9.18 mm, 9.32 mm, and 9.57 mm, respectively. Similarly, mean PFH was 8.15 mm, 8.30 mm, and 8.52 mm, respectively. There was a significant difference in HVID, VVID, and PFH among the three age groups (*P* ≤ 0.001), as well as among male and female children in the 6–10 years age group (*P* ≤ 0.05) but not in the other groups. Intraclass correlation coefficient values (0.78–0.95) show good agreement among the three optometrists for all parameters. The normal range of HVID, VVID, and PFH presented in the current study can help practitioners in the diagnosis of corneal disorders, serve as a basis for the design of contact lenses, and enable accurate intraocular lens power calculations for South Indian children.

## INTRODUCTION

The measurement of corneal diameter (CD) is based on the visible iris, and it has two components. The horizontal corneal diameter (horizontal visible iris diameter, HVID) is measured as the distance between the nasal and temporal imaginary tangents to the corneal circumference along the center of the pupil. The vertical corneal diameter (vertical visible iris diameter, VVID) is measured as the distance between the superior and inferior imaginary tangents to the corneal circumference [1]. CD is important in clinical settings, being vital in ensuring that the total diameter of a soft lens is sufficient to maintain full corneal coverage [2]. The importance of deviations from normal values in the diagnosis of ocular anomalies, such as relative anterior microphthalmos, microcornea, and congenital glaucoma, makes the measurement of CD particularly relevant in pediatric ophthalmology [3–5], and it is also useful in determining the size of intraocular lenses [6,7]. Palpebral fissure height (PFH), measured as the vertical distance between the open eyelids, is crucial for ocular prostheses and essential for measuring ptosis [8]. Both CD and PFH have an important role in the selection of contact lens parameters [9,10].

CD and PFH may be influenced by variables such as age, sex, and ethnicity [1,11–17]. Although there is a vast literature regarding the reference values of CD and PFH among different races in the adult population, limited data exist in the pediatric population, especially in the South Indian region. Hence, the aim of this study was to measure these parameters in a cohort of South Indian children and provide a reference database for clinicians.

In routine clinical practice, CD is frequently assessed using a hand-held millimeter ruler, caliper, or the graticule of a slit lamp [5,10]. Advanced instruments such as optical coherence tomography, auto-refractometer, and corneal topographers can also be used to measure CD accurately [18]. However, these instruments are not available in all clinics and require the child’s cooperation. A millimeter ruler is easily accessible and enables a simple and quick measurement. We consider that the normative data measured with the help of a ruler would aid ophthalmologists and optometrists in their clinical practice. Therefore, the current study aimed to establish normative values of CD and PFH in school-aged children.

## MATERIAL AND METHODS

### Study design

We carried out a prospective, cross-sectional study that involved school children chosen at random from six schools in two areas in Tamil Nadu state of South India.

### Inclusion and exclusion criteria

The study included children aged 4–15 years, with no history of eye surgery or systemic diseases, with refractive errors within ± 6D, who were co-operative for eye examination, normal anterior segment with torchlight examination, and normal red reflex in both eyes on direct ophthalmoscopy examination.

Children with a history of systemic disorders, endocrine diseases, or ocular surgery, as well as refractive errors larger than 3D were excluded from the study. Children with ocular tumors, orbital deformities, buphthalmos, craniofacial anomalies, extraocular muscle palsy, or nystagmus were also excluded.

### Study population and measurements

Children were divided by age into three groups, pre-primary children (4–5 years), primary school children (6–10 years), and high school children (11–15 years). Each child underwent screening for visual acuity, objective refraction, torch light examination, relative afferent pathway defect (RAPD), and red reflex test using direct ophthalmoscopy.

**Figure 1:**
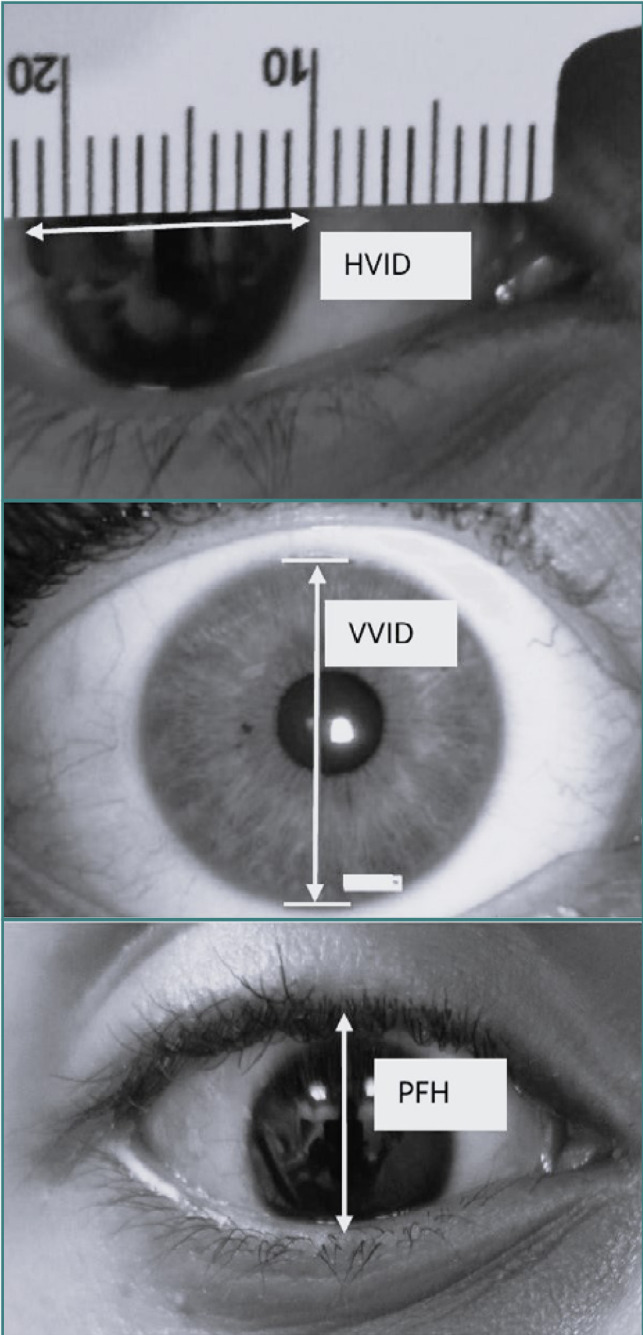
HVID, VVID, and PFH measurements in the study groups

HVID, VVID, and PFH were measured three times by a trained optometrist using a simple millimeter ruler and avoiding parallax error (Figure 1). The average of the three measurements was considered the final value of that parameter in each subject. The three parameters were measured by at least one optometrist in all children and by three optometrists in 20% of the children.

### Statistical analysis

The collected data were entered in Microsoft Excel sheets and then transferred to SPSS v.25.0 (IBM Corp). Descriptive analysis was used to report the mean HVID, VVID, and PFH values among the groups. The Pearson correlation coefficient was used to report the association between age and ocular parameters. A statistically significant difference (*P* ≤ 0.05) in HVID, VVID, and PFH values between male and female children was determined using an independent *t*-test. The variance of HVID, VVID, and PFH values among the different age groups was reported using analysis of variance (ANOVA), and the Bonferroni post-hoc correction was applied to determine which groups exhibited statistically significant differences. Intraclass correlation coefficients (ICC) were used to report the variability among the measured values by each examiner, and also the variability of the measured values among the examiners. ICC estimations and their 95% confidence intervals were obtained using an absolute-agreement, two-way mixed-effect model.

## RESULTS

A total of 1,258 children were selected for the study, 24 of which were uncooperative and we could not obtain data from them. The final study population included 1,234 children, 621 of which were male (50.32 %) and 613 were female (49.68 %). The mean age of the entire sample was 9.43 ± 3.4 years, and the mean age of male and female children was 9.39 ± 0.61 years and 9.47 ± 0.62 years, respectively, with no statistically significant difference (*P* = 0.07). The mean age of preschool children was 4.49 ± 0.50 years, of primary school children 8.00 ± 1.41 years, and of high school children 12.87 ± 1.42 years. Given that there was a strong statistically significant correlation (*P* < 0.001) between the right and left eye parameter values of the children (*r =* 0.93 for HVID, *r =* 0.97 for VVID, and *r =* 0.92 for PFH), only right eye data is reported in the analysis. Descriptive statistics of HVID, VVID, and PFH for both eyes of all study subjects according to age group are summarized in Table 1.

**Table 1:** HVID, VVID, and PFH of right eye (OD) and left eye (OS) among the three age groups. Data are expressed as mean ± s.d.

Age group	*n*	HVID OD (mm)	HVID OS (mm)	VVID OD (mm)	VVID OS (mm)	PFH OD (mm)	PFH OS (mm)	Age (years)
**4–5 years**	207	10.45 ± 0.37	10.41 ± 0.35	9.18 ±0.35	9.16 ±0.36	8.15 ± 0.67	8.09 ± 0.65	4.49 ± 0.50
**6–10 years**	517	10.54 ± 0.43	10.56 ± 0.43	9.32 ± 0.49	9.30 ± 0.49	8.30 ± 0.71	8.29 ± 0.70	8.00 ± 1.41
**11–15 years**	510	10.73 ± 0.36	10.72 ± 0.36	9.58 ± 0.48	9.55 ± 0.48	8.53± 0.65	8.53 ± 0.65	12.87 ± 1.42
**Total**	1,234	10.60 ± 0.41	10.60 ± 0.41	9.41 ±0.49	9.38 ± 0.49	8.37 ± 0.70	8.36 ± 0.69	9.43 ± 3.40

### Effect of age on ocular parameters

**Figure 2:**
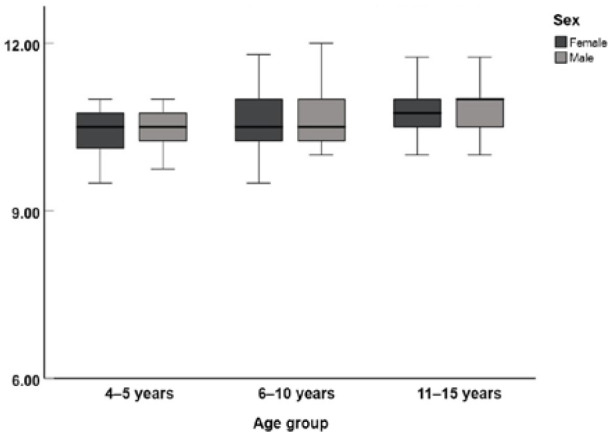
Box plots of HVID according to age and sex

Box plots of the mean right eye HVID for the three age groups are shown in Figure 2. The mean HVID measurements for the right eye for children aged 4–5 years, 6–10 years, and 11–15 years were 10.45 ± 0.37 mm, 10.54 ± 0.43 mm, and 10.73 ± 0.36 mm, respectively.

**Figure 3:**
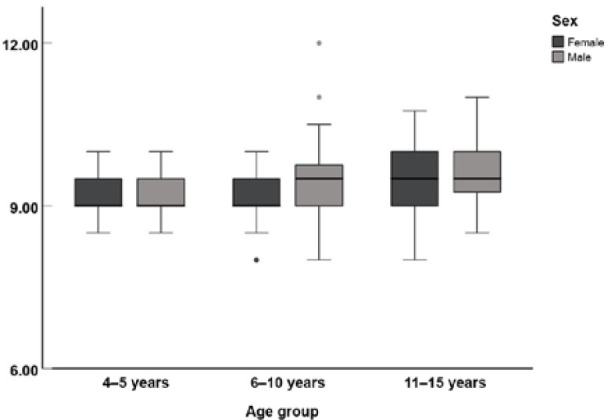
Box plots of VVID according to age and sex. Circular grey and black circles are the outliers.

Box plots of the mean right eye VVID for the three age groups are shown in Figure 3. The mean VVID measurements for the right eye for children aged 4–5 years, 6–10 years, and 11–15 years were 9.18 ± 0.35 mm, 9.32 ± 0.49 mm, and 9.57 ± 0.48 mm, respectively.

**Figure 4:**
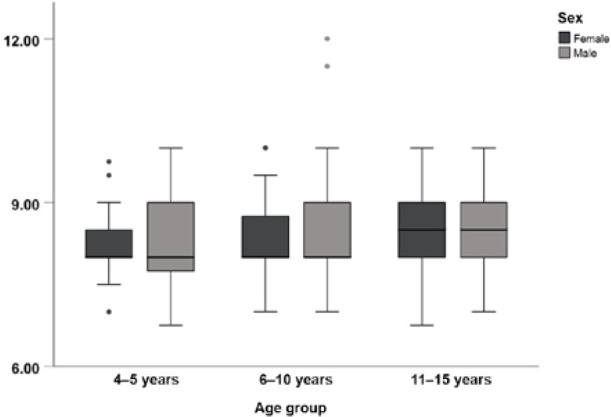
Box plots of PFH according to age and sex. Circular grey and black circles are the outliers.

Box plots of the mean right eye PFH for the three age groups are shown in Figure 4. The mean PFH measurements for the right eye for children aged 4–5 years, 6–10 years, and 11–15 years were 8.15 ± 0.67 mm, 8.32 ± 0.71 mm, and 8.52 ± 0.65 mm, respectively.

There was a significant difference in the mean values of HVID, VVID, and PFH among the three age groups (*P* ≤ 0.001) (Table 2).

**Table 2:** Mean HVID, VVID, and PFH values in the three age groups

Parameter	Age group	*n*	Mean	s.d.	*P* value
**HVID OD (mm)**	4–5 years	207	10.45	0.37	≤0.001
	6–10 years	517	10.55	0.44	
	11–15 years	510	10.73	0.38	
	Total	1,234	10.60	0.42	
**VVID OD (mm)**	4–5 years	207	9.18	0.36	≤0.001
	6–10 years	517	9.32	0.49	
	11–15 years	510	9.58	0.48	
	Total	1,234	9.40	0.49	
**PFH OD (mm)**	4–5 years	207	8.15	0.67	≤0.001
	6–10 years	517	8.30	0.71	
	11–15 years	510	8.52	0.66	
	Total	1,234	8.37	0.70	

### Effect of sex on ocular parameters

The mean ocular parameters among male and female children for the three age groups are presented in Table 3. There was no significant difference (*P* > 0.05) in mean HVID and VVID values between male and female children in the 4–5 years and 11–15 years age groups, but there was a significant difference (*P* ≤ 0.05) between these parameters in the 6–10 years age group. By contrast, there was a statistically significant difference in mean PFH values between sexes in the 4–5 years and 6–10 years age group (*P* ≤ 0.05) but not in the 11–15 years age group.

**Table 3:** Comparison of mean ocular parameters by sex

Parameter	Age group	Male	Female	*P* value
**HVID OD (mm)**	4–5 years	10.45 ± 0.35	10.44 ± 0.40	0.169
	6–10 years	10.58 ± 0.47	10.51 ± 0.40	0.03*
	11–15 years	10.75 ± 0.38	10.72 ± 0.38	0.27
	Total	10.63 ± 0.42	10.58 ± 0.42	0.57
**VVID OD (mm)**	4–5 years	9.20 ± 0.36	9.16 ± 0.36	0.976
	6–10 years	9.38 ± 0.52	9.27 ± 0.43	0.03*
	11–15 years	9.59 ± 0.46	9.56 ± 0.49	0.36
	Total	9.43 ± 0.49	9.37 ± 0.49	0.98
**PFH OD (mm)**	4–5 years	8.13 ± 0.74	8.16 ± 060	0.06
	6–10 years	8.35 ± 0.78	8.24 ± 0.64	0.01*
	11–15 years	8.49 ± 0.67	8.56 ± 0.65	0.78
	Total	8.37 ± 0.73	8.26 ± 0.66	0.01*

* Statistically significant

### Intraobserver and interobserver variability

Three examiners (optometrists) have measured the ocular parameters of the study population. Intraobserver variability based on intraclass correlation coefficients (ICCs) is presented in Table 4. ICC values were in the range of 0.61–0.97, suggesting good reliability between the measurements. Similarly, interobserver variability is reported using ICCs in Table 5. ICC values were in the range of 0.78–0.95, suggesting that there is very good agreement in measuring the ocular parameters among the three examiners using the millimeter ruler.

**Table 4:** Interobserver variability among the three examiners

Parameter	ICC		95% CI	*P* value
**HVID**	Single measure	0.78	0.75–0.81	<0.0001^*^
	Average measure	0.92	0.90–0.93	
**VVID**	Single measure	0.87	0.85–0.89	
	Average measure	0.95	0.94–0.96	
**PFH**	Single measure	0.89	0.87–0.91	
	Average measure	0.94	0.93–0.95	

* Statistically significant

**Table 5:** Intraobserver variability among the three examiners

Examiner	Parameter	ICC		95% CI	*P* value
**Examiner 1**	HVID	Single measure	0.90	0.87–0.93	<0.0001^*^
		Average measures	0.97	0.96–0.98	
	VVID	Single measure	0.94	0.91–0.96	
		Average measures	0.98	0.98–0.99	
	PFH	Single measure	0.88	0.83–0.91	
		Average measures	0.97	0.95–0.98	
**Examiner 2**	HVID	Single measure	0.61	0.51–0.71	
		Average measures	0.86	0.81–0.91	
	VVID	Single measure	0.65	0.56–0.74	
		Average measures	0.88	0.83–0.92	
	PFH	Single measure	0.78	0.72–0.84	
		Average measures	0.94	0.94–0.91	
**Examiner 3**	HVID	Single measure	0.89	0.85–0.92	
		Average measures	0.97	0.96–0.98	
	VVID	Single measure	0.94	0.92–0.96	
		Average measures	0.98	0.97–0.99	
	PFH	Single measure	0.87	0.83–0.91	
		Average measures	0.97	0.95 – 0.98	

* Statistically significant

## DISCUSSION

The current study reports HVID, VVID, and PFH values from 1,234 healthy Indian children of various age groups. Mean HVID and VVID values among different races are presented in Table 6. CD values measured in this study in the 6–10 years age group are lower than those reported in Malaysian, Chinese, and Brazilian studies [11,19–23]. Earlier studies have shown that there was a significant difference in HVID between male and female children [20,23]. However, we found a statistically significant difference in CD values between male and female children only in the 6–10 years age group, and we were not able to attribute a specific reason for this difference.

**Table 6:** Mean HVID, VVID, and white-to-white CD values among different populations in the literature

Author & year	Population	Age group (years)	*n*	HVID (mm)	VVID (mm)	WTW CD (mm)	Instrument
Chan *et al*., 2011 [11]	Chinese	6–12	217	11.3 ± 0.3 male 11.3 ± 0.3 female	–	–	Medmont E300 Topographer
Ali *et al*., 2011 [19]	Malaysian	7–12 13–18	188 196	11.89 ± 0.36 11.92 ± 0.29	11.29 ± 0.27 11.23 ± 0.34	–	Auto refractometer
Jiang *et al*., 2017 [20]	Chinese	4–18	5,970	12.02 ± 0.38	–	–	Laser interferometer
Costa *et al*., 2005 [21]	Brazilian	4–5 5.1–6.5	17 14	11.96 ± 0.33 12.07 ± 0.42	–	–	Caliper
Wang *et al*., 2019 [22]	Chinese	5–18	48	–	–	11.66 ± 1.92	IOL master
Zhao *et al*., 2023 [23]	Chinese	4–9	1,528	–	–	12.08 ± 0.43 male 11.94 ± 0.44 female	IOL master
Current study	Indian	4–15	1,234	10.61 ± 0.42	9.40 ± 0.49	–	Millimeter ruler

WTW, white-to-white

Mean HVID ranged from 10.45 mm to 10.73 mm in our study, which is lower than the values reported for an adult Indian population with a mean HVID of 11.74 ± 0.32 mm [14], and higher than the values reported for a group of Indian newborns with a mean HVID of 9.5 ± 0.6 mm [24]. These findings suggest that in the Indian cohort corneal diameters increase from birth to adulthood, but in this study mean PFH values varied considerably between the three groups and increased with age.

Mean PFH values ranged from 8.15 mm to 8.52 mm in the three age groups, being smaller than those reported for a group of Malaysian children [19] and larger than those reported for Korean [25] and Chinese [26] children. Cai *et al*. found that PFH values increased from childhood to adulthood in a Chinese population and suggested that several factors may affect the PFH, including the development of the craniofacial complex, the levator muscle, the tarsal plates, and the epicanthus, as well as changes in the skin and elastic fibers around the eye [26]. We strongly assume that the growth of the levator muscle may be the main reason for the increase in PFH from childhood to adulthood.

The control of myopia has been a primary reason for teenagers to wear contact lenses, especially in Asian countries [11], as properly designed contact lenses can halt the progression of myopia [27,28]. Given the importance of corneal characteristics in contact lens design [29,30], accurate knowledge of children’s corneal profiles is crucial to the success of contact lens fittings. However, there is limited information on the corneal profiles of Indian children. One important measure for contact lens fitting is HVID. Compared to individuals of European descent, those from South East Asia typically have lower HVID and smaller palpebral aperture sizes. Most lenses currently available on the market are larger and designed based on data from European and American populations. As a result, these lenses can pose problems such as difficulty with insertion and removal, discomfort, and vision-related issues. Owing to the increasing popularity of contact lenses among young individuals and the availability of more complex contact lens designs, manufacturers may need consider variations in corneal features when producing lenses for young populations. The data presented in this study may provide valuable reference values for manufacturers to create contact lenses for Indian children.

The main limitation of this study is that HVID, VVID, and PFH were measured using a simple ruler, and the results are expressed in 1-mm steps. Compared to alternative methods, such as Orbscan, digital photography, and calipers, which can measure these characteristics to the nearest 0.1 mm, a millimeter ruler results in more measurement inaccuracy. However, we believe that a simple millimeter ruler is more practical and available to all practitioners dealing with ocular metrics, including ophthalmologists, optometrists, and other subspecialties. ICC values showed good agreement between the average measurements of different examiners, suggesting that a simple millimeter ruler is sufficiently accurate to measure these parameters, effectively serving the practitioner’s purpose.

## CONCLUSION

The normal range of HVID, VVID, and PFH presented in the current study can help practitioners in the diagnosis of ocular disorders such as megalocornea, microcornea, and microphthalmos. They can also serve as a basis for the future design of contact lenses for children aged 4–15 years of Indian origin, and aid ophthalmic surgeons in intraocular and implantable collamer lens power calculations.

## Data Availability

Further data is available from the corresponding author on request.
